# Supplementary material for: Evolution of service metrics and utilisation of objective discharge criteria in anterior cruciate ligament reconstruction rehabilitation: a retrospective cohort study with historical control in a public hospital physiotherapy department
Source: Arch Physiother. 2020 Dec 14;10:23. doi: 10.1186/s40945-020-00093-9 (PMC7737268; doi:10.1186/s40945-020-00093-9)
Supplement: Supplementary file 1 — Additional file 1: Phases, Outcome Measures and Discharge Criteria. [file 40945_2020_93_MOESM1_ESM.pdf]

Appendix 1. Phases, Outcome Measures and Discharge Criteria

Phase number	Phase focus	Phase goals
1	Recovery	▪ Manage pain and swelling<br>▪ Achieve knee extension<br>▪ Begin quadriceps activation
2	Strength and neuromuscular control	▪ Full knee ROM<br>▪ No knee swelling<br>▪ Regain single leg balance<br>▪ Regain most of muscle strength<br>▪ Good lower limb control with single leg squat
3	Running, agility and landings	▪ Maximise muscle strength<br>▪ Excellent jump/re-bound technique<br>▪ Excellent single leg landing control<br>▪ Return to running<br>▪ Good control straight line agility
4	Return to sport	▪ Increase power activity<br>▪ Excellent control with change of direction agility tasks<br>▪ Increase confidence in knee<br>▪ Complete sport-specific tasks
5	Prevention	▪ Maintain strength, power, and neuromuscular control<br>▪ Prevent knee re-injury

Outcome Measure	Goal for Discharge
Single leg squat x 5	> 60° depth, smooth, good alignment
Single Leg Press 1RM (kg)	>90% LSI
Single Leg Bridge (max reps)	>90% LSI
Single Leg Calf Raise (max reps)	>90% LSI
Side Plank (time to fatigue)	>90% LSI
SEBT (cm)	>95% LSI
Single Hop for Distance (cm)	>90% LSI
Triple Cross-Over Hop (cm)	>90% LSI

LSI - Limb Symmetry Index
