# Supplementary material for: Evolution of service metrics and utilisation of objective discharge criteria in anterior cruciate ligament reconstruction rehabilitation: a retrospective cohort study with historical control in a public hospital physiotherapy department
Source: Arch Physiother. 2020 Dec 14;10:23. doi: 10.1186/s40945-020-00093-9 (PMC7737268; doi:10.1186/s40945-020-00093-9)
Supplement: Supplementary file 2 — Additional file 2: Clinical practice: models of care. [file 40945_2020_93_MOESM2_ESM.pdf]

Appendix 2. CLINICAL PRACTICE: MODELS OF CARE

	**Pre Packaged Model of Care**	**Post Packaged Model of Care - 1:1**	**Post Packaged Model of Care - ACL group**
**Therapist-Patient Interaction**	1:1 therapist to patient	1:1 therapist to patient	Group setting (up to 5:2 ratio)
**Appointment duration**	Up to 30 mins 1:1	Up to 30 mins 1:1	1 hour
**Exercise selection**	As per surgeon protocol followed by clinician choice	Phase guided exercise program targeting neuromuscular control, stability, strength and agility<br><br>*clinician able to tailor for patient	Phase guided exercise program targeting neuromuscular control, stability, strength and agility<br><br>*clinician able to tailor for patient<br><br>One hour available to complete prescribed exercise program while under physiotherapist supervision
**Objective monitoring & phase progression**	Clinician guided	Assessment guided by evidence-based protocol, including strength and functional outcome measures<br><br>Clear objective goals to achieve to progress through rehabilitation	Assessment guided by evidence-based protocol, including strength and functional outcome measures<br><br>Clear objective goals to achieve to progress through rehabilitation
**Discharge process**	Clinician individual judgement or timeframe post-surgery	Use of discharge criteria to ensure minimise risks of RTS<br><br>Clear education regarding ongoing self-management to minimise risks of re-injury	Use of discharge criteria to ensure minimise risks of RTS<br><br>Clear education regarding ongoing self-management to minimise risks of re-injury
